# A Case Report on an Elusive Incident of Erythema Multiforme

**DOI:** 10.21980/J8BM0W

**Published:** 2025-01-31

**Authors:** Cynthia Tsang, Savannah Tan, Lindsey Spiegelman

**Affiliations:** * University of California, Irvine, School of Medicine, Irvine, CA; ^ University of California, Irvine, Department of Emergency Medicine, Orange, CA

## Abstract

The presentation of erythema multiforme in the emergency department is relatively rare, thus recognition and rapid intervention requires a high index of suspicion. This study presents a case of a 55-year-old female with past medical history of hypertension and active endometrial cancer with recent chemotherapy treatment complaining of four days of progressive erythematous rash with associated pruritis and blistering. An exam found multiple tense, scattered vesicles with an erythematous base. The patient also demonstrated leukopenia, elevated alkaline phosphatase level, and elevated C-reactive protein level. A shave biopsy was performed and intravenous acyclovir was started for concern of varicella-zoster virus. Biopsy results favored an erythema multiforme diagnosis, and she was discharged with topical clobetasol. In addition to reviewing the presentation and intervention of erythema multiforme, this case report adds to growing literature of erythema multiforme as a delayed reaction to malignancy therapy.

**Topics:**

Erythema multiforme, dermatology, radiotherapy.

**Figure:**
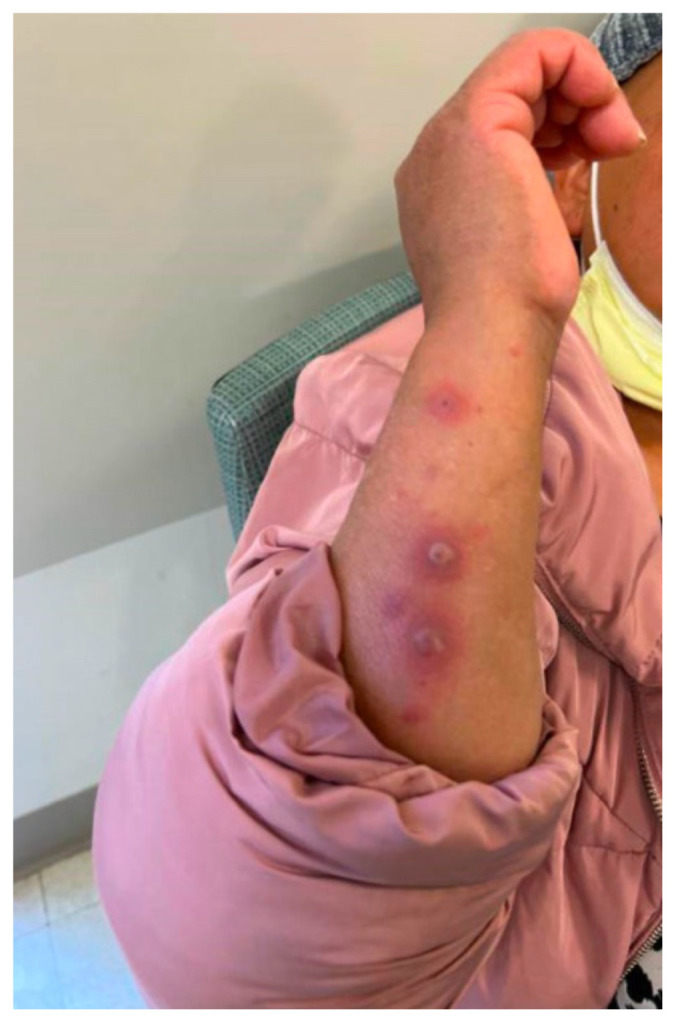


**Figure:**
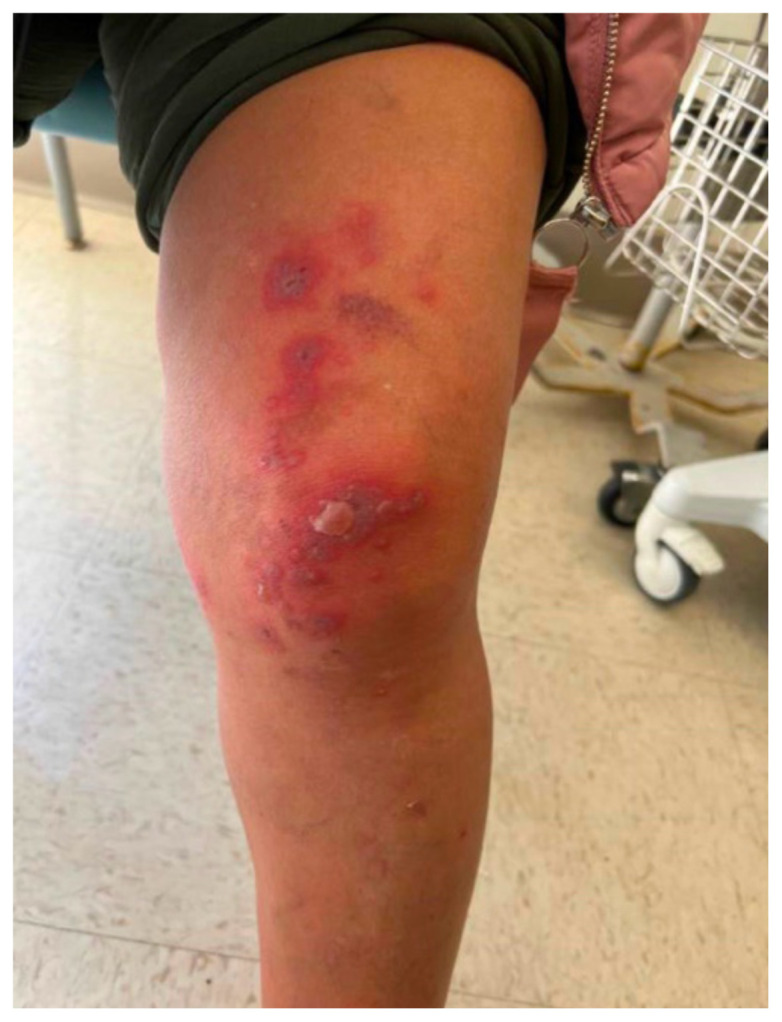


**Figure:**
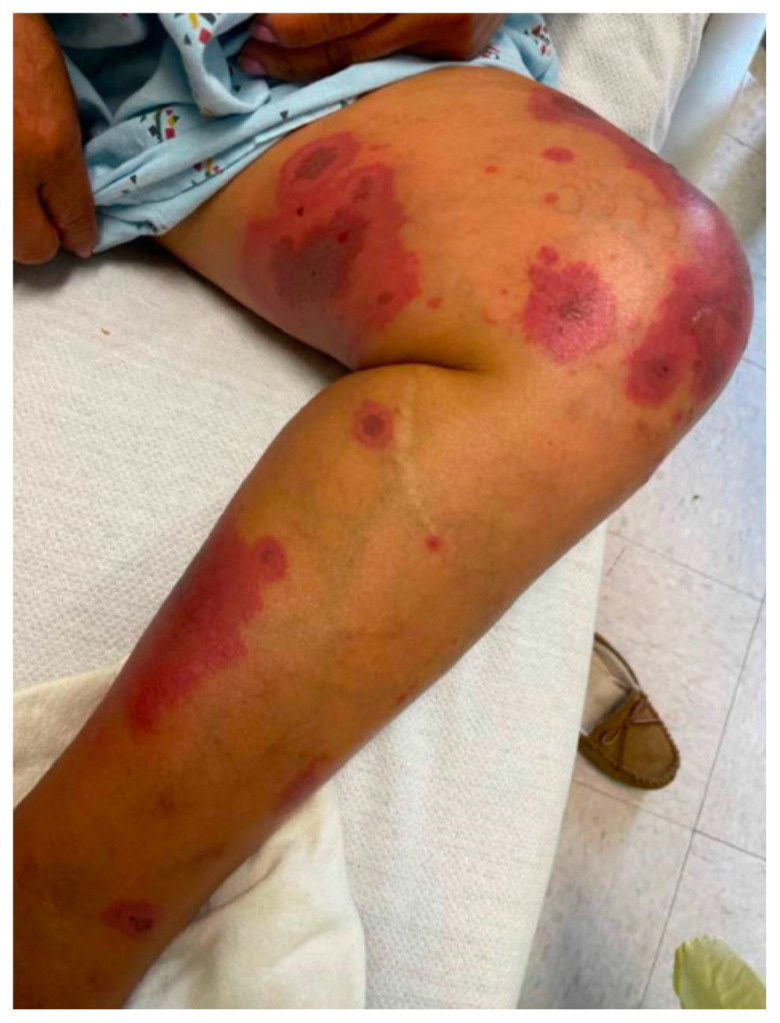


## Brief introduction

Erythema multiforme (EM) is an acute, cutaneous, and mucosal type IV hypersensitivity reaction commonly occurring in response to infection or medications, including chemotherapeutic agents.^1^,^2^,^3^ EM can be further divided into minor and major forms.^4^,^5^ The classic or minor variety refers to EM with little to no mucosal symptoms and is typically caused by herpes simplex virus (HSV).^2^ The major form presents with severe mucosal involvement and systemic effects, typically in response to medications.^2^ Imitators of EM include Stevens-Johnson syndrome (SJS), toxic epidermal necrolysis (TEN), urticaria, and bullous pemphigoid.^1^ The annual incidence of EM is estimated at <1%, occurring more commonly in adults younger than 40.^3^,^6^,^7^ While most cases of EM are solitary events, patients may experience recurrent EM.

Hallmark EM target lesions begin as burning or itching pink or red papules that then become plaques.^8^ A conventional lesion has a center with concentric regions of color change, typically presenting on extremities.^9^ Aside from skin or mucosal lesions, initial EM symptoms can also include fever, fatigue, headache, and sore throat. Because the presentation of EM has significant overlap with other dermatological and systemic reactions, it is important to understand the varied ways and populations in which this condition can present to further delineate diseases and describe nuances. We discuss EM in a 55-year-old female patient with endometrial cancer and explore her unique case in association with recent intervention for malignancy. Awareness of EM presentation and progression may inform future protocols and improve timeliness of patient care. Written consent for publication was obtained from the patient.

## Presenting concerns and clinical findings

A 55-year-old immunocompromised female with a past medical history significant for hypertension, anemia, and active endometrial cancer with history of recent chemotherapy presented to the emergency department (ED) complaining of a spreading rash that began developing four days prior. The rash started in her left lower extremity with pruritis and a burning sensation, then spread to her right lower extremity and right upper extremity. Around this time, blisters began to form in those same regions. The patient tried over-the-counter hydrocortisone which did not improve the symptoms. She noticed worsening of the lesions with scratching. The patient denied fever, chills, and oral lesions. She had not started any new medications, lotions, soaps, or detergents prior to symptom onset. Of note, her last infusion of carboplatin-taxol was five months prior to presentation. The differential diagnoses included contact dermatitis, atopic dermatitis, disseminated herpes zoster, and erythema multiforme. Less likely diagnoses included SJS, TEN, bullous pemphigoid, and pemphigus vulgaris.

## Significant findings

The patient’s initial vital signs included a temperature of 98.2°F, respiratory rate of 20, a mildly elevated blood pressure of 151/82, a heart rate of 94, and a blood oxygen saturation of 99% on room air. Her physical exam was notable for multiple scattered tense vesicles on an erythematous base along the left and right lower extremities and right upper extremity. The lesions were excoriated and in different stages of evolution. No oral, mucosal, or conjunctival lesions were found. Physical exam was otherwise unremarkable.

The patient’s complete blood count was without anemia but showed leukopenia (2.6 thous/uL) which could be explained by her ongoing chemotherapy treatment course. An elevated alkaline phosphatase level was stable from prior lab work completed one month prior to her presentation. C-reactive protein was elevated at 1.1 mg/dL.

## Patient course

Dermatology was consulted and performed a shave biopsy of lesions on the right forearm. A lesion swab was also collected to test for varicella-zoster virus. Out of concern for disseminated herpes zoster infection, dermatology recommended admitting the patient and initiating empiric intravenous acyclovir 10 mg/kg every eight hours for the next seven to ten days. The patient was admitted the same day for suspected disseminated herpes zoster. During admission, diphenhydramine 25 mg was given every six hours as needed to alleviate skin pruritis. Airborne and contact precautions were also taken while waiting for biopsy and serology results.

The biopsy showed no viral cytopathic features suggestive of herpes virus infection and instead favored an erythema multiforme diagnosis. Given that the patient was undergoing active cancer treatment, had manageable symptoms, and developed few new lesions during her hospital stay, she was deemed stable for discharge. Upon discharge, dermatology recommended starting topical clobetasol 0.05% ointment twice daily to affected areas, transitioning to oral acyclovir while awaiting the final varicella-zoster virus culture result, and follow up with outpatient dermatology in two to four weeks. The patient’s varicella-zoster virus polymerase chain reaction (PCR) eventually resulted as negative, and acyclovir was discontinued.

## Discussion

Erythema multiforme is a cutaneous or mucocutaneous condition that initially presents with multiple skin papules in the extremities that can progress to plaques. The etiology of EM can be drug-induced or triggered by HSV infection, though the condition can be seen in patients with lupus erythematosus,^10^ polymorphic light eruption,^11^ atypical pneumonia,^12^ HIV,^13^ adult T cell lymphoma,^14^ and more. EM can be subclassified as minor or major depending on mucosal involvement. In this patient, biopsy of the lesion confirmed EM with little to no mucosal involvement, so she was diagnosed with the minor variant. Because EM, particularly EM minor, has an indistinct initial manifestation, diagnosis is most commonly based on clinical appearance. For confirmation and definitive diagnosis, providers can order a skin biopsy.

This case review adds to growing literature that draws associations between EM and malignancy intervention. One previous study characterized EM-like rashes that developed during or shortly after thirty patients received radiotherapies.^15^ All patients experienced a disseminated, pink-red edematous papular rash that began at the site of irradiation, spread with time, and started a median of 19.5 days after radiation. Sixty percent of patients (18 participants) recovered from topical corticosteroids alone, while 30% (ten participants) required both topical corticosteroids and oral antihistamines to resolve symptoms. There was a low rate of recurrent EM, with only 13% (four participants) developing a second episode. Interestingly, in this cohort of thirty, many of the rashes developed along the trunk, though EM has canonically favored extremities. Cases of radiation-associated skin reactions consistently defy this paradigm, with lesions that begin at the irradiation site.^3^ Another case report by Ambur et al^16^ demonstrates EM rashes following combination pembrolizumab and radiation for stage IV non-small cell lung cancer in a patient who presented with eroded plaques surrounded by targetoid and vesicular lesions five weeks post-intervention.^16^ They describe how further radiation events are linked to episodes of recurrent EM in the trunk and extremities. Han et al^17^ reported a patient with rectal malignancy treated with 5-fluorouracil radiotherapy only to present with EM pruritic targetoid papules and confluent macules on the buttocks, dorsum of the hands, and the ears six months after treatment.^17^ The patient began a fifth cycle of chemotherapy and EM recurred in extremities. Another case report described a patient undergoing radiation therapy for large cell lung carcinoma that developed EM two weeks after completing his last series of treatments which started on the dorsum of his hands and spread to his palms, forearms, and chest.^18^ These studies vary in many ways in malignancy type, organ system, and chemotherapeutic agent, but they all share a similar delayed EM reaction. The onset of an EM rash appears between two weeks and six months post-treatment, a range our patient, who received her last infusion five months prior to presentation, falls within. Reports also illustrate not only higher rates of EM recurrence is patients receiving cancer interventions, but EM episodes that temporally follow treatments with symptoms that resolve after radiation termination. These studies help contextualize this patient’s presentation, correlating her recent chemotherapy treatment with the onset of dermatological symptoms in the ED.

It is essential to know that induction of EM following combination radiation-drug therapy is an established phenomenon. Erythema multiforme associated with phenytoin and cranial radiation therapy (EMPACT) is an accepted and expected response in patients who undergo radiotherapy of the head and receive prophylactic anticonvulsant therapy, a standard of care for intracranial malignancies.^19^,^20^,^21^ Studies have shown that 8.5–19% of patients can develop EM reactions an average of 23 days post-radiation.^19^ Any cutaneous reactions in this population is closely evaluated with a high index of suspicion for EM, because instances of demise are not uncommon.^22^,^23^,^24^ Like EM reactions in patients receiving chemotherapeutic agents, the EMPACT rash begins at the irradiation site, and is successfully managed with cessation of offending drug and consistent topical corticosteroids use until EM resolution.^23^

Multiple case reports have been written on EM reactions following radiation treatment, though no large clinical studies have been conducted on this subject. A study on 151 evaluable cases found that EM, SJS and TEN were rarely reported in patients that had radiotherapy alone; rather, a majority of the cases occurred in irradiated patients that were simultaneously treated with pharmacologic therapies.^25^,^26^ A short list of drugs, including antiepileptics like phenytoin or phenobarbital, are known to incite EM episodes, but to our knowledge there have been no reports of carboplatin-taxol induced EM in the literature.

This case report describes a patient with recent history of chemotherapeutic infusion five months prior to presentation who was ultimately diagnosed with erythema multiforme minor. Appropriate therapy was delayed out of concern for disseminated herpes zoster infection, and diagnosis was established with skin biopsy. While EM-induced by carboplatin-taxol or other chemotherapeutic agents has not been widely studied, this case and the expanding number of reports it contributes to offer a frontier worthy of exploring with larger cohorts and greater granularity. For clinicians who would be this patient’s first line provider, documentation of this case may elucidate more instances of EM following carboplatin-taxol treatment to ensure early recognition of this condition and prompt treatment.

## Supplementary Information






