# Supplementary figures and images for: A Case Report on an Elusive Incident of Erythema Multiforme
Source: J Educ Teach Emerg Med. 2025 Jan 31;10(1):V17–21. doi: 10.21980/J8BM0W (PMC11801485; doi:10.21980/J8BM0W)

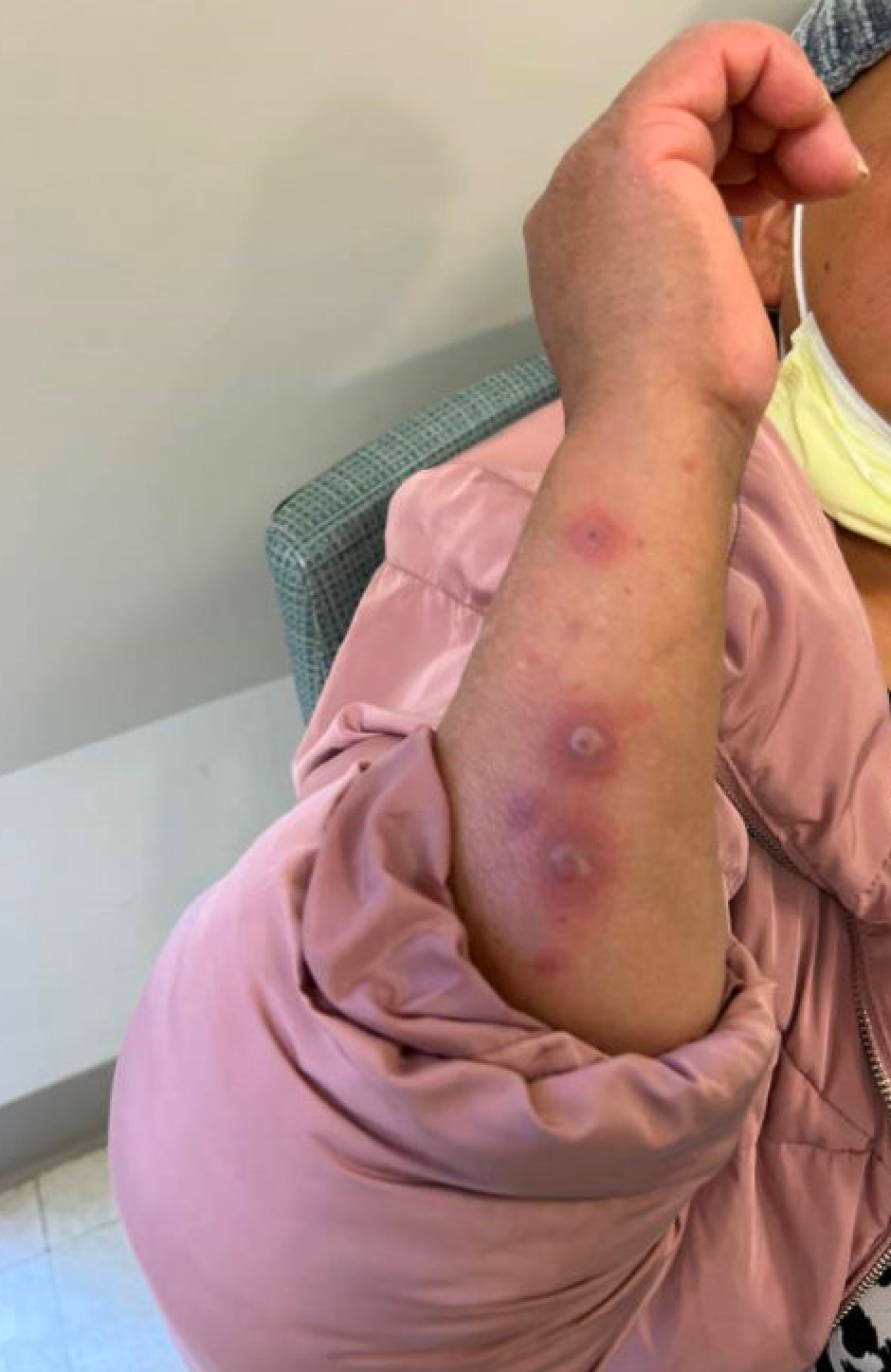

Supplement: Supplementary file 1 [file 10-1-V17-supp1.jpeg]

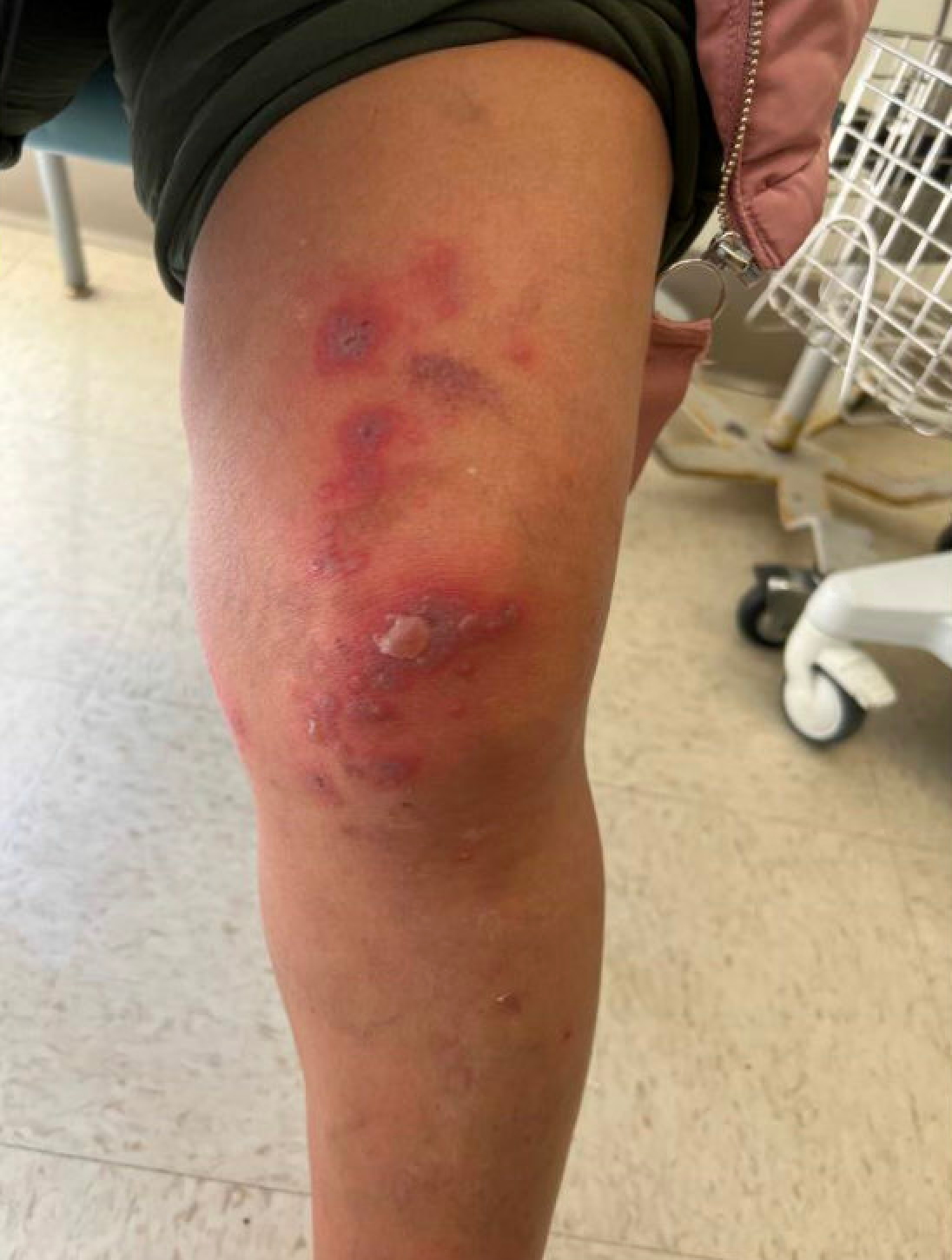

Supplement: Supplementary file 2 [file 10-1-V17-supp2.jpeg]

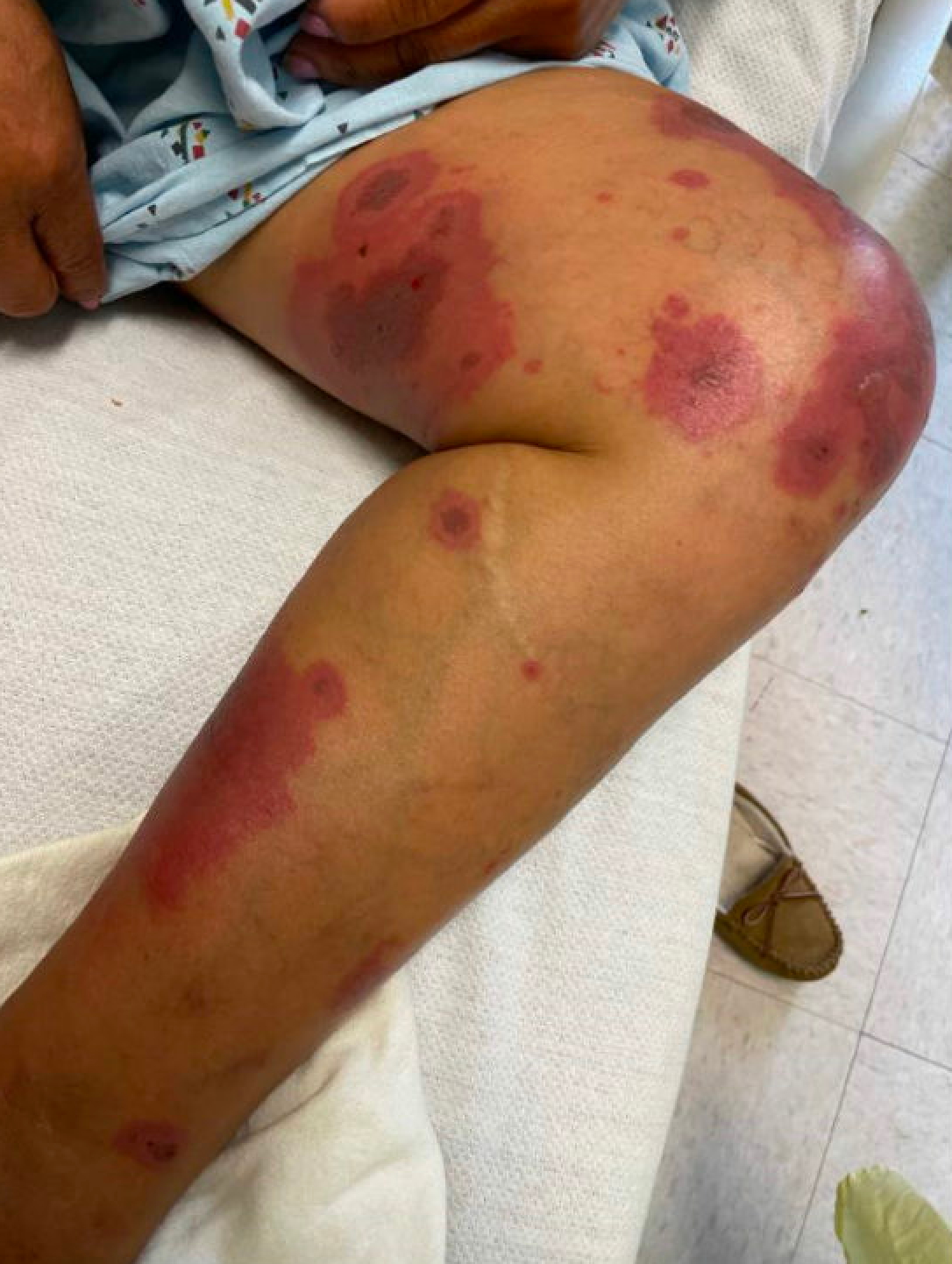

Supplement: Supplementary file 3 [file 10-1-V17-supp3.jpeg]
